# Hepatic FoxO1 Integrates Glucose Utilization and Lipid Synthesis through Regulation of Chrebp O-Glycosylation

**DOI:** 10.1371/journal.pone.0047231

**Published:** 2012-10-08

**Authors:** Yukari Ido-Kitamura, Tsutomu Sasaki, Masaki Kobayashi, Hye-Jin Kim, Yong-Soo Lee, Osamu Kikuchi, Hiromi Yokota-Hashimoto, Katsumi Iizuka, Domenico Accili, Tadahiro Kitamura

**Affiliations:** 1 Metabolic Signal Research Center, Institute for Molecular and Cellular Regulation, Gunma University, Maebashi, Gunma, Japan; 2 Division of Molecule and Structure, Department of Diabetes and Endocrinology, Gifu University School of Medicine, Gifu, Gifu, Japan; 3 Department of Medicine, College of Physicians and Surgeons of Columbia University, New York, New York, United States of America; 4 Department of Internal Medicine, Fujisawa Shounandai Hospital, Fujisawa, Kanagawa, Japan; Joslin Diabetes Center-, Harvard Medical School, United States of America

## Abstract

In liver, glucose utilization and lipid synthesis are inextricably intertwined. When glucose availability exceeds its utilization, lipogenesis increases, leading to increased intrahepatic lipid content and lipoprotein secretion. Although the fate of three-carbon metabolites is largely determined by flux rate through the relevant enzymes, insulin plays a permissive role in this process. But the mechanism integrating insulin receptor signaling to glucose utilization with lipogenesis is unknown. Forkhead box O1 (FoxO1), a downstream effector of insulin signaling, plays a central role in hepatic glucose metabolism through the regulation of hepatic glucose production. In this study, we investigated the mechanism by which FoxO1 integrates hepatic glucose utilization with lipid synthesis. We show that FoxO1 overexpression in hepatocytes reduces activity of carbohydrate response element binding protein (Chrebp), a key regulator of lipogenesis, by suppressing O-linked glycosylation and reducing the protein stability. FoxO1 inhibits high glucose- or O-GlcNAc transferase (OGT)-induced liver-pyruvate kinase (L-PK) promoter activity by decreasing Chrebp recruitment to the L-PK promoter. Conversely, FoxO1 ablation in liver leads to the enhanced O-glycosylation and increased protein level of Chrebp owing to decreased its ubiquitination. We propose that FoxO1 regulation of Chrebp O-glycosylation is a mechanism linking hepatic glucose utilization with lipid synthesis.

## Introduction

The liver plays a central role in integrating glucose and lipid metabolism, effectively exchanging carbons from one energy source to the other for storage and utilization [1]. This process requires both hormone signaling and feedback control by substrate flux. Examples of the latter are the diversion of three-carbon precursors from glycolysis to esterification of FFA to generate triglycerides, and the shunting of citrate from glycolysis to fatty acid synthesis by way of acetyl-CoA carboxylase and malonyl-CoA [2]. Examples of the former are the effects of insulin on expression of genes that rate-control glucose utilization and its conversion into lipids, like glucokinase, glucose-6-phosphatase, pyruvate kinase, and pyruvate dehydrogenase kinase [1]. In physiological situations, the two control mechanisms cohabit peacefully. But in the metabolic syndrome, there is an apparent discrepancy between the inability of insulin to suppress glucose production, and its preserved ability to promote de novo lipogenesis. Various theories have been advanced, but none of them is entirely satisfactory [3].

Key transcriptional mediators of insulin signaling and glucose signaling are FoxO1 and Chrebp. FoxO1 is an Akt substrate and regulates glucose production and bile acid synthesis [4]–[5]. Chrebp mediates glucose action on glycolysis and lipid synthesis [6]. Among its targets are liver-pyruvate kinase (*Lpk*), one of the rate-limiting enzymes of glycolysis [7], and lipogenic genes, such as acetyl-CoA carboxylase (*Acaca*) and fatty acid synthase (*Fasn*) [8]. Chrebp is activated via protein phosphatase 2A-dependent dephosphorylation in response to xylulose-5-phosphate (Xu-5-P) generated by the pentose monophosphate shunt [9]. Dephosphorylated Chrebp translocates to the nucleus and activates target gene transcription [10].

Impetus for the present studies came from prior observations that genetic ablation of FoxO1 in liver increases systemic insulin sensitivity, and results in lower hepatic glucose production, increased glycogen storage, and increased lipogenesis [11]–[12]. We reasoned that this model could be deconstructed for the purpose of identifying the physiological mechanism linking glucose with lipid metabolism. We identify a genetic, biochemical, and molecular pathway linking FoxO1 with Chrebp, and propose that it represents the connection between altered glucose and lipid metabolism in type 2 diabetes.

## Methods

### Antibodies

We purchased antibodies against Chrebp from Novus Biologicals, O-GlcNAc from Covance, OGT (DM-17) from Sigma, FoxO1 (9462) from Cell Signaling, FoxO1 (H-128), Ubiquitin (P4D1), Tubulin (B–7) from Santa-Cruz, HA (12CA5) from Roche. We used these antibodies for immunoprecipitation or immunoblotting according to manufacturer's protocol.

### Expression vectors and Adenoviral vectors

We have previously described expression vectors encoding Flag-tagged FoxO1-ADA and His-HA-Ubiquitin [13], and adenoviral vectors encoding HA-tagged wild type and FoxO1-ADA [14]. pcDNA3-HA-OGT and pCMV4-Flag-Chrebp are gifts from Mark Montminy (Salk Institute, La Jolla, CA) and Howard Towle (Univ of Minnesota, Minneapolis, MN), respectively. We generated a synthetic L-PK luciferase vector containing 3x charbohydrate response element in the L-PK promoter (pGL3-3xL-PK-ChoRE).

### Cell culture, siRNA transfection, and viral transduction

We purchased primary culture of mouse hepatocyte from Primary Cell Co., Ltd (Sapporo, Japan) and cultured the cells in DMEM supplemented with 10% FCS. The FoxO1-specific siRNA sequence is 5′-ACGGAGGATTGAACCAGTATA-3′. The OGT specific siRNA sequence is 5′-CGACATGCCTTGCGGCTGA -3′. siRNA was transfected using DharmaFECT Duo (Dharmacon). In some experiments, we infected primary hepatocytes with adenovirus at MOI of 10 or 30, 5 hrs before treatments with high glucose. All experiments were repeated at least three times.

### Luciferase assays and Chromatin immunoprecipitation assays

We performed luciferase assays as previously described [15] using pGL3-3xL-PK-ChoRE. We performed ChIP assays in mouse primary hepatocytes or mouse liver extracts with primers; 5′-GATTTGAGCCTTTGATCCAGGCTC-3′ and 5′-AAGTTCCCTCCATCTATACAGTGC-3′ according to the previously described methods [13]. All experiments were repeated at least three times.

### Immunoprecipitation and Western blotting

We lysed cultured cells in RIPA buffer containing protease inhibitors (Roche). After centrifugation, cell extracts were diluted with Co-IP buffer (50 mM Tris, 150 mM NaCl, 0.1% NP-40, 10% glycerol, 5 mM MgCl_2_), immunoprecipitated and analyzed by immunoblotting.

### mRNA isolation and real-time PCR

We isolated mRNA from primary hepatocytes or mouse liver extracts using the Micro Fast Track 2.0 kit (Invitrogen). We performed real-time RT-PCR using ImProm-II™ Reverse Transcription System (Promega) and LightCycler System (Roche). Primer sequences used for real-time PCR are as follows, for Chrebp; 5′- CTG GGG ACC TAA ACA GGA GC -3′ and 5′- GAA GCC ACC CTA TAG CTC CC −3′, for L-PK; 5′- GGG CCG CAT CTA CAT TGA C −3′ and 5′- GTC CCT CTG GGC CAA TTT T-3′. We carried out each reaction in triplicate, using a standard curve with the relevant cDNA for each primer set.

### O-GlcNAc enzymatic labeling

We performed metabolic labeling of Chrebp with tetraacetylated azide-modified N-acetylglucosamine (GlcNAz) in mouse primary hepatocytes. After immunoprecipitation with anti-Chrebp antibody, we detected O-glycosylation modification using biothin-avidin system.

### Animal generation and analyses

We generated liver specific FoxO1 knockout mice using FoxO1 flox/flox mice [16] and Albumin-cre transgenic mice (a kind gift from Akihiro Harada, Osaka University). The wild-type, null and *Foxo1^flox^* alleles were detected using PCR with primers 5′-GCT TAG AGC AGA GAT GTT CTC ACA TT-3′, 5′-CCA GAG TCT TTG TAT CAG GCA AAT AA-3′ and 5′-CAA GTC CAT TAA TTC AGC ACA TTG A-3′. Individually caged mice were housed in a temperature-controlled facility. All animal care and experimental procedures were approved by the Institutional Animal Care and Experimentation Committee at Gunma University. H-E staining was performed using 4- µm-thick paraffin sections following the standard methods. Hepatic triglyceride (TG) contents were measured as described previously [17].

## Results

### FoxO1 inhibits Chrebp transcriptional activity by suppressing O-glycosylation and reducing protein stability of Chrebp

**Figure 1:**
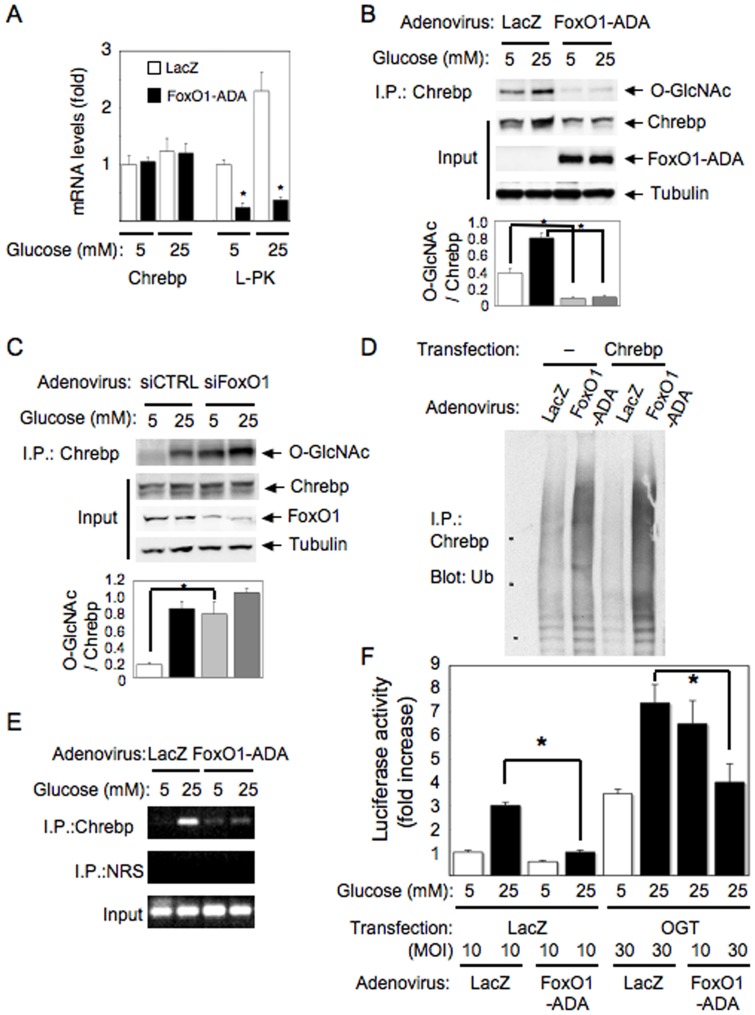
FoxO1-ADA expression and the knockdown of FoxO1 affect Chrebp O-glycosylation, protein stability and transcriptional activity in primary hepatocytes. (A, B, D and E) Mouse primary hepatocytes infected with adenovirus expressing FoxO1-ADA or LacZ were cultured with 5 mM or 25 mM glucose. The cell lysates were subjected to real-time RT-PCR for Chrebp or L-PK (A), immunoprecipitation with anti-Chrebp antibody followed by blotting with anti-O-GlcNAc antibody (B) or anti-ubiquitin antibody (D). The cell lysates were also subjected to chromatin-immunoprecipitation assay using anti-Chrebp antibody and the primers for L-PK promoter (E). (C) Mouse primary hepatocytes infected with adenovirus expressing specific siRNA for FoxO1 or control siRNA were cultured with 5 mM or 25 mM glucose and the cell lysates were immunoprecipitated with anti-Chrebp antibody followed by blotting with anti-O-GlcNAc antibody. Input represents expression levels of Chrebp, FoxO1-ADA, endogenous FoxO1 and Tubulin. Quantitative analyses were performed by assessment of O-glycosylation level compared with the protein level of Chrebp using densitometry, showing as a bar graph below the results of blotting (B and C). (F) Mouse primary hepatocytes co-transfected with pGL3-3Xl-PK-ChoRE and OGT or LacZ were infected with adenovirus expressing FoxO1-ADA or LacZ at indicated MOI and cultured with 5 mM or 25 mM glucose for 24 hr. The cell lysates were used for luciferase assays. Experiments were repeated at least three times. Data represent mean ± SEM. *P<0.05.

Although insulin as well as glucose flux regulate hepatic glucose utilization and lipid synthesis, the underlying molecular mechanisms have not been fully understood. Because FoxO1 is a downstream effector of insulin signaling and Chrebp is a key transcriptional regulator of glycolysis and lipogenesis, we tested whether FoxO1 is involved in the regulation of Chrebp. When we overexpressed constitutively active form of FoxO1, FoxO1-ADA (a mutant FoxO1 with the following amino acid substitutions: T24A, S253D, and S316A) [18], in primary hepatocytes, mRNA level of *Lpk*, a target of Chrebp, was significantly decreased, despite unchanged levels of *Chrebp* mRNA (Fig. 1A). Therefore, we next investigated Chrebp protein levels in these samples. As shown in Figure 1B, FoxO1-ADA expression significantly decreased Chrebp protein level (Fig. 1B, second panel from the top). Because it has been reported that Chrebp protein is stabilized by modification of O-glycosylation [19], we investigated it and found that FoxO1-ADA expression decreased Chrebp O-glycosylation (Fig. 1B, top panel and the bottom graph). Consistent with the previous report [19], O-glycosylation and protein level of Chrebp were increased by high glucose (25mM) in primary hepatocytes (Fig. 1B). Conversely, when we knocked down FoxO1 using an adenovirus expressing FoxO1-specific siRNA in primary hepatocytes, Chrebp O-glycosylation was increased independent of glucose concentrations in the medium (Fig. 1C, top panel and the bottom graph).

Because protein level, but not mRNA level of Chrebp was decreased by FoxO1-ADA, we next checked whether FoxO1-ADA expression affects protein degradation of Chrebp by evaluating its ubiquitination. As shown in Fig. 1D, FoxO1-ADA expression enhanced poly-ubiquitination of both endogenous (left two lanes) and exogenously expressed Chrebp (right two lanes), indicating that changes in Chrebp ubiquitination might explain the decreased protein levels observed in our experiments. Indeed, chromatin immunoprecipitation revealed that overexpression of FoxO1-ADA suppressed high-glucose-induced recruitment of Chrebp to the *Lpk* promoter (Fig. 1E). Furthermore, results of luciferase assays using the *Lpk* promoter indicated that FoxO1-ADA inhibited high-glucose-induced or OGT-expression-induced Chrebp transcriptional activity (Fig. 1F). Taken together, these results show that FoxO1 inhibits Chrebp transcriptional activity by suppressing O-glycosylation and reducing protein stability of Chrebp.

### Chrebp O-glycosylation is regulated by OGT

**Figure 2:**
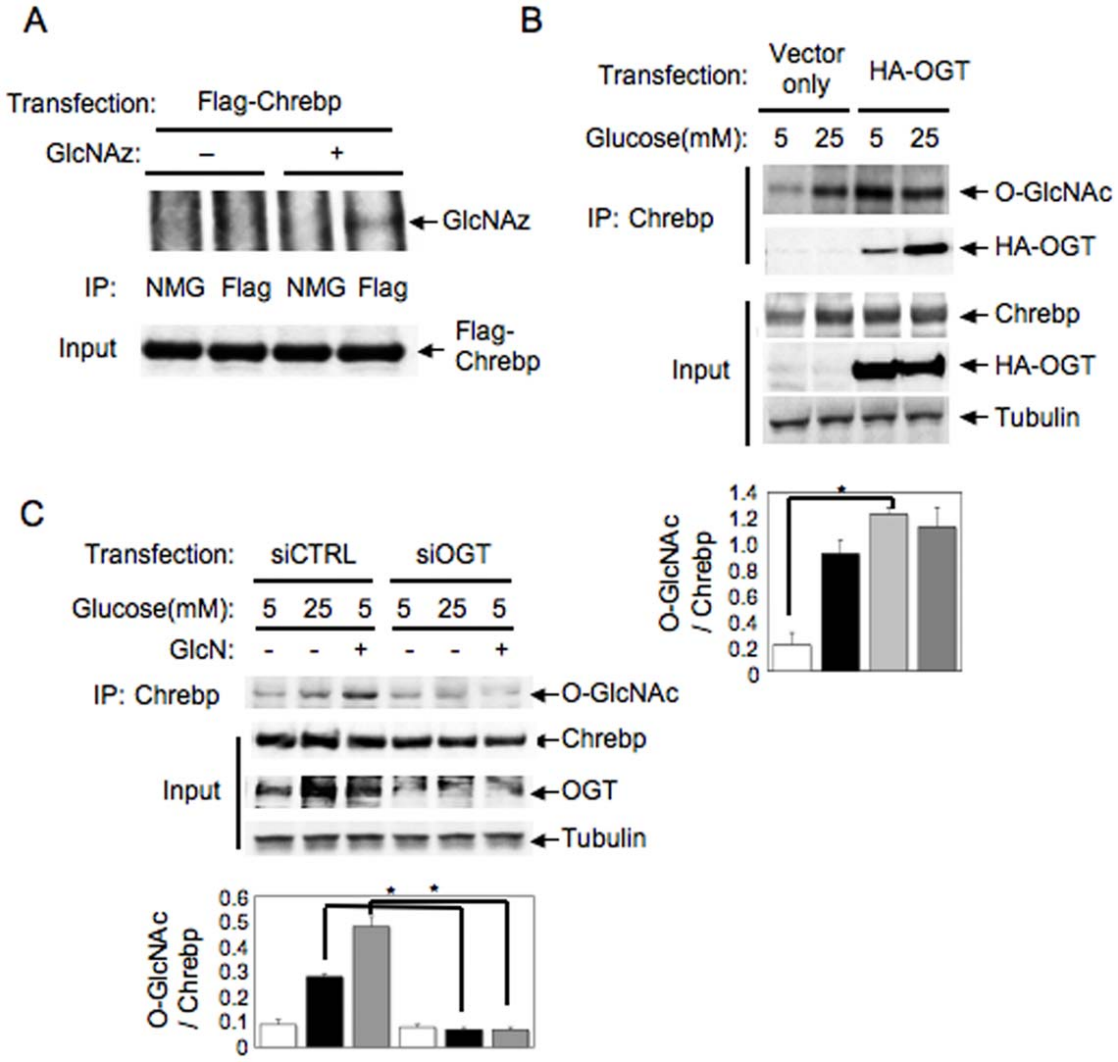
Chrebp O-glycosylation is regulated by OGT. (A) Mouse primary hepatocytes were transfected with Flag-Chrebp and labeled with tetraacetylated azide-modified N-acetylglucosamine (GlcNAz). The cell lysates were immunopricipitated with anti-Flag antibody or normal mouse globlin (NMG) and subjected to detection of O-glycosylation by biothin-avidin system as described in Materials and Methods. Input indicates the expression level of Flag-Chrebp in each lane. (B and C) Mouse primary hepatocytes were transfected with HA-OGT (B) or siRNA for OGT (C) along with empty vector or control siRNA, respectively and cultured with 5 mM or 25 mM glucose for 24 hr. In some experiments, 10 mM glucosamine (GlcN) was added in the medium. The cell lysates were immunoprecipitated with anti-Chrebp antibody followed by blotting with anti-O-GlcNAc or anti-HA antibody. Input indicates the expression level of Chrebp, HA-OGT, endogenous OGT or tubulin. Quantitative analyses were performed by assessment of O-glycosylation level compared with the protein level of Chrebp using densitometry, showing as a bar graph below the results of blotting (B and C). Experiments were repeated at least three times. Data represent mean ± SEM. *P<0.05.

We used western blotting with anti-GlcNAc antibody to show that Chrebp is O-glycosylated in the presence of elevated glucose concentrations (Fig. 1B). To confirm the modification of Chrebp O-glycosylation, we also used enzymatic labeling with GlcNAz in primary hepatocytes. As shown in Figure 2A, we detected GlcNAz incorporation into Chrebp protein only in the presence of GlcNAz, indicating that Chrebp is directly modified by O-glycosylation. We next investigated whether high glucose-induced Chrebp O-glycosylation is mediated by OGT. When we transfected primary hepatocytes with OGT, Chrebp O-glycosylation was significantly increased even in low glucose condition (Fig. 2B, top panel and bottom graph). It is notable that OGT was coimmunoprecipitated with Chrebp, and that this interaction was enhanced by high glucose (Fig. 2B, second panel from the top). Conversely, when OGT was depleted by specific siRNA for OGT in primary hepatocytes, both high glucose- and glucosamine-induced O-glycosylation of Chrebp were decreased (Fig. 2C, top panel and bottom graph). Thus, Chrebp interacts with OGT in high glucose condition, leading to Chrebp O-glycosylation.

### Increased Chrebp O-glycosylation, protein stability, and recruitment to the Lpk promoter in FoxO1 knockout liver

**Figure 3:**
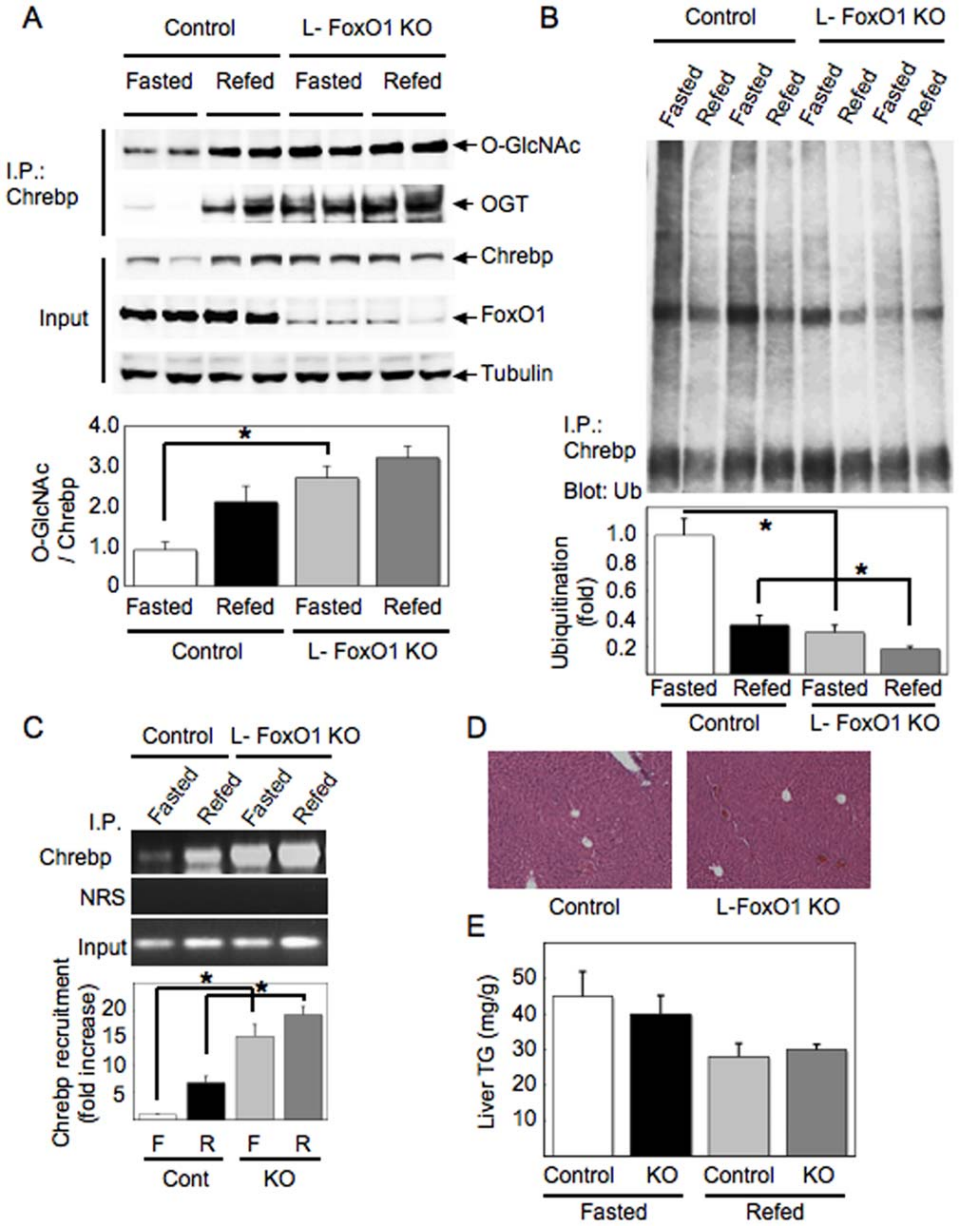
Chrebp O-glycosylation, protein stability, and recruitment to the L-PK promoter are increased in FoxO1 knockout liver. (A and B) The liver samples isolated from L-FoxO1-KO and the control mice at the points of 24 hr fasted or 3 hr refed after 24 hr starvation were subjected to immunoprecipitation with anti-Chrebp antibody followed by blotting with anti-O-GlcNAc, anti-OGT (A) or anti-ubiquitin antibody (B). Input indicates the expression levels of Chrebp, FoxO1 or tubulin. Quantitative analyses were performed by assessment of O-glycosylation (A) or ubiquitination (B) levels compared with the protein level of Chrebp using densitometry, showing as a bar graph below the results of blotting. (C) The liver extracts were also subjected to chromatin immunoprecipitation assay using anti-Chrebp antibody and primers for the L-PK promoter. Quantitative analyses were performed using densitometry. Input indicates extracted DNA prior to immunoprecipitation. Experiments were repeated at least three times. Data represent mean ± SEM. *P<0.05. (D) Hematoxylin-and-eosin (H-E) staining of the liver sections from 24 hr fasted L-FoxO1-KO and the control mice. Magnification, x100. (E) Hepatic triglyceride (TG) contents in 24 hr fasted or 3 hr refed (after 24 hr starvation) L-FoxO1-KO and the control mice (n = 6 for each group). Data represent mean ± SEM.

To assess the effects of FoxO1 ablation in liver on Chrebp protein, we generated liver-specific FoxO1 knockout mice (L-FoxO1-KO) by crossing Albumin-Cre mice with FoxO1 flox mice [16]. We isolated livers from L-FoxO1-KO and control mice following a 24-hr fast or 3-hr re-feeding after 24-hr fast, and performed western blotting or ChIP assays using liver lysates. In the liver of L-FoxO1-KO mice, Chrebp O-glycosylation and protein levels were significantly increased, and–more interestingly–OGT interaction with Chrebp was enhanced in both fasted and refed conditions compared with the liver of control mice (Fig. 3A, top two panels). Consistent with the increase in Chrebp protein level, Chrebp poly-ubiquitination were significantly reduced in the liver of L-FoxO1-KO mice in both fasted and refed conditions (Fig. 3B). We also found that Chrebp recruitment to the *Lpk* promoter was enhanced in the liver of L-FoxO1-KO mice compared with controls (Fig. 3C), consistent with our previous results showing that *Lpk* mRNA was increased in liver-specific FoxO1 knockout mice [11].

We next investigated the physiological consequences of FoxO1 ablation in the liver. Because we showed FoxO1 ablation in the liver enhanced Chrebp protein stability and Chrebp recruitment to its target gene promoter, we predicted that hepatic lipid contents should be increased in L-FoxO1-KO mice. However, histological analysis using the liver sections showed no morphological difference between L-FoxO1-KO and control mice (Figure 3D). Furthermore, hepatic triglyceride contents were unchanged in L-FoxO1-KO mice in both fasted and refed conditions (Figure 3E).

## Discussion

Our studies identify a direct molecular link between insulin signaling pathways regulating hepatic glucose production and those regulating glycolysis and lipogenesis. In addition to Chrebp, another critical transcription factor for lipogenesis is sterol regulatory element-binding protein 1c (Srebp-1c) [20]. Although the transcriptional activity of Srebp-1c is mainly regulated by the cleavage of its NH_2_-terminal domain and nuclear translocation [21], it is also known that Srebp-1c is regulated by insulin at the transcriptional level via liver X receptor (LXR) [22]. Recently, it has been shown that Chrebp, like Srebp-1c, is a direct target of LXR [23], indicating that Chrebp may be also regulated at the transcriptional level by insulin. Our data demonstrate a different mechanism of regulation, as we show that protein, but not mRNA levels of Chrebp are regulated by various metabolic conditions in primary hepatocytes and mouse liver. We also show that Chrebp is O-glycosylated by high glucose (in hepatocytes) and re-feeding (in liver), leading to increased protein level of Chrebp, owing to decreased ubiquitination.

Glucose taken into hepatocytes is mainly converted to pyruvate or glycogen to produce or store energy. However, excess glucose enters into hexosamine biosynthetic pathway (HBP), leading to the production of UDP-N-acetylglucosamine (UDP-GlcNAc). By using UDP-GlcNAc as the donor substrate, O-GlcNAc transferase (OGT) catalyzes O-glycosylation modification of proteins on Ser/Thr residues. Although only ∼2–3% of intracellular glucose enters the HBP [24], it is known that hyperglycemia increases glucose flux into HBP and subsequent O-glycosylation of various proteins [25]. Furthermore, transgenic mice overexpressing OGT show diabetic phenotype due to insulin resistance [26]. Taken together, these data suggest that the increase in O-glycosylation is associated with the pathophysiology of diabetes. Recently, three key transcription factors for glucose metabolism, FoxO1, Pgc-1α and Torc2 (Crtc2) have been reported to be regulated by O-glycosylation modification [27]–[30]. Furthermore, Guinez et al. reported that Chrebp is also regulated by O-glycosylation, leading to the increase in Chrebp protein level and its transcriptional activity [19].

**Figure 4:**
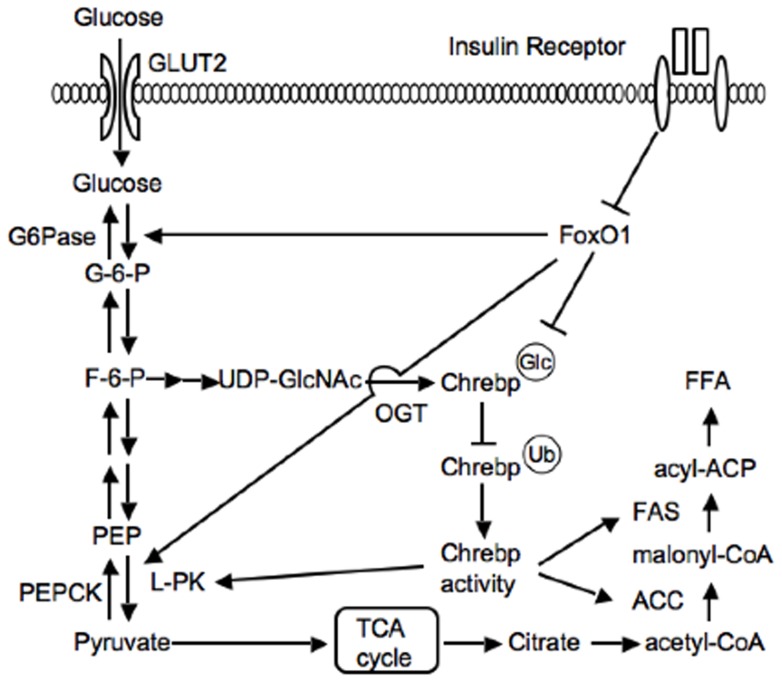
Proposed model for the role of FoxO1 integrating glucose utilization and lipid synthesis through regulation of Chrebp O-glycosylation.

FoxO1 is a member of the forkhead box containing protein of the O subfamily, which regulates metabolism as well as cellular proliferation, apoptosis, differentiation and stress resistance [4]. FoxO1 transcriptional activity is regulated by insulin through phosphorylation by Akt and following nuclear exclusion [31]–[33]. We previously reported that FoxO1 plays a central role in regulating glucose production in liver through the regulation of gluconeogenic genes, such as glucose-6-phosphatase (G6Pase) and phosphoenolpyruvate-carboxykinase (PEPCK) [34] [12]. We also previously reported that hepatic FoxO1 ablation leads to slight increases in *Fasn* and *Lpk*, two critical Chrebp targets, without affecting *Chrebp* mRNA levels [11]. Thus, in addition to the function of FoxO1 to increase gluconeogenesis, we propose here that FoxO1 also decreases glucose utilization and lipid synthesis by reducing Chrebp activity. Because insulin essentially inhibits FoxO1 transcriptional activity through nuclear exclusion, insulin increases glucose utilization and lipid synthesis as well as decreases glucose production (Fig. 4).

In this study, we showed FoxO1 ablation in the liver enhanced Chrebp protein stability and Chrebp recruitment to its target gene promoter. Therefore we predicted that hepatic lipid synthesis should be increased in L-FoxO1-KO mice. However, hepatic lipid contents were unchanged in these mice (Fig. 3D and 3E). One possible explanation for this phenotype was that because hyperglycemia-induced oxidative stress leads to FoxO1 activation by acetylation-dependent mechanism as we previously reported [13], the effect of FoxO1 on lipid metabolism might only become apparent in hyperglycemic conditions. Another explanation was that because not only Chrebp but also Srebp1c or LXR contribute to the regulation of hepatic lipid metabolism, the effect of FoxO1 ablation might be compensated by the other factors *in vivo*.

Considering that different amino acid residues are targeted by O-glycosylation (Ser/Thr) *vs*. ubiquitination (Lys), it remains unclear how increased O-glycosylation is associated with decreased ubiquitination of Chrebp. However, one possible mechanism is that O-glycosylation may change protein structure, affecting the susceptibility of ubiquitination and subsequent protein degradation [35]. In future studies, it will be of importance to unveil the mechanism by which FoxO1 inhibits Chrebp O-glycosylation.
